# Home-based low-level light therapy using a commercial LED mask for dry eye disease

**DOI:** 10.1007/s10792-026-04219-z

**Published:** 2026-08-29

**Authors:** Luca Scuderi, Serena Fragiotta, Chiara Ciancimino, Gianluca Scuderi

**Affiliations:** 1 Department of Sense Organs, University of Rome Sapienza, 00161 Rome, Italy; 2 Ophthalmology Unit, Neurosciences, Mental Health, and Sensory Organs (NESMOS) Department, Sapienza University of Rome, Via Di Grottarossa 1035/1039, 00189 Rome, Italy

**Keywords:** Dry eye syndrome, Low-level light therapy (LLLT), Breakup time (TBUT), Schirmer’s test, Tear osmolarity

## Abstract

**Purpose:**

Light-based therapies, including low-level light therapy (LLLT), have recently emerged as promising non-invasive approaches for dry eye disease (DED). This study evaluates the impact of a consumer-grade home-based LED Beauty Mask on DED parameters.

**Methods:**

This retrospective observational cohort study included 20 postmenopausal women (20 eyes) diagnosed with moderate DED and MGD who underwent cosmetic treatment using the LED Beauty Mask Koled®. The device was used for 20-min sessions on alternate days over a 3-week period. Clinical evaluations were performed at baseline, 3 weeks (T1), and 2 weeks (T2) after the last treatment and included tear osmolarity, tear film break-up time (TBUT), Schirmer I test, corneal fluorescein staining, and the Ocular Surface Disease Index (OSDI).

**Results:**

Tear osmolarity demonstrated a significant improvement over time (*P* < 0.001), with the most pronounced change observed at T1. Tear film stability, assessed by TBUT, also improved significantly (*P* = 0.001). Subjective symptoms measured by OSDI showed a significant and sustained reduction (*P* < 0.001). No significant changes were observed in Schirmer I test values (*P* = 0.29). Corneal fluorescein staining present at baseline resolved by the final follow-up. Four eyes (20%) exhibited osmolarity values below the measurable range after treatment. No discomfort or adverse events were reported.

**Conclusions:**

This study suggests that a cosmetic LED mask may provide symptomatic and functional improvement in DED when repurposed for ocular use. Although preliminary and limited by sample size, these findings support further investigation through controlled clinical trials.

## Introduction

Dry eye disease (DED) is a common ocular surface disorder, affecting approximately 14–33% of the global population and has a considerable impact on both quality of life and workplace productivity [1]. It is characterized by tear film instability, hyperosmolarity, ocular surface inflammation, factors, which together create a ‘vicious circle’ [2]. Several treatment strategies have been developed in recent years to target inflammation and tear film components of DED. These strategies include tear film supplementation through artificial tears, anti-inflammatory and immunomodulatory treatments, secretagogues and stimulation of endogenous tear production, and biologic and regenerative agents [3].

More recently, light-based therapies, including intense pulsed light (IPL) and low-level light therapy (LLLT), have emerged as novel biomodulation techniques for the treatment of DED, particularly in cases associated with Meibomian gland dysfunction (MGD). IPL therapy involves the application of non-coherent, polychromatic light to the periorbital region, typically delivered from tragus to tragus. Its therapeutic effects include reducing reactive oxygen species (ROS), modulating inflammation, decreasing telangiectatic vessels, suppressing matrix metalloproteinases (MMPs), stimulating fibroblast activity and collagen synthesis, and eradicating *Demodex* mites [4, 5].

LLLT utilizes light-emitting diodes (LEDs) to deliver specific red or near-infrared wavelengths of light directly to the tissues, activating cellular chromophores and stimulating mitochondrial function, notably the production of adenosine triphosphate (ATP) [4, 6]. This cellular photostimulation promotes tissue repair and functional improvement.

Among the available technologies, Light Modulation® LLLT [7, 8] (Meibomask®) is the only patented system designed specifically for ophthalmic use. It employs red light at 630 nm to stimulate Meibomian gland activity, improve the lipid layer of the tear film, and relieve symptoms of DED. Its clinical applications include treatment of MGD, blepharitis, chalazion, Sjögren’s syndrome, and post-surgical dry eye [7–11]. Despite its demonstrated efficacy and excellent safety profile, widespread clinical adoption remains limited due to high equipment costs and logistical burdens, both for healthcare providers and patients.

However, the widespread use of consumer-grade LED home devices, initially developed for dermatological and cosmetic use, may have potential ophthalmic applications. One such device is the LED Beauty Mask Koled® (https://koled.it), an ergonomically designed mask equipped with 576 LEDs covering the facial and periocular region. It operates at multiple wavelengths, including red light at 630 nm—the same wavelength used in standard LLLT protocols—and offers the advantage of home-based use at a significantly lower cost.

Given the technical similarities between the LED Beauty Mask Koled® and professional LLLT systems, and considering its lower cost and greater accessibility, it may serve as a practical and scalable alternative for the management of dry eye syndrome.

The present study aimed to report a cohort of patients with DED undergoing dermatological treatment with the LED Beauty Mask Koled® and to assess the potential impact of this dermatological treatment on dry eye symptoms.

## Materials and methods

This was a retrospective observational cohort study including 20 postmenopausal women (mean age 63.4 ± 3.2 years) diagnosed with moderate DED and MGD. All patients were undergoing regular follow-up for DED at the Academic Center–Sant’Andrea Hospital, Rome, and sought evaluation regarding the potential effects of the device on their ocular surface status between March and December 2025. Independently of their clinical care, the patients had obtained the LED Beauty Mask Koled® for elective cosmetic anti-aging use. Patients were neither required nor advised to acquire the device for clinical treatment of DED, as this use is not currently considered standard of care. The study received approval from the Institutional Review Board (IRB) and adhered to the tenets of the Declaration of Helsinki. All subjects provided written informed consent to participate in the study.

DED was diagnosed according to the TFOS DEWS II criteria [12]. Patients were diagnosed with DED if they had dry eye symptoms assessed through the Ocular Surface Disease Index (OSDI) and at least one abnormal dry eye test [12, 13], as summarized in Table 1.

**Table 1:** Tear film and ocular surface society dry eye workshop II (TFOS DEWS) diagnostic criteria

Diagnostic criteria	Tests	Cut-off values
Dry eye symptoms	OSDI	≥ 13/100
Tear film stability	Tear film break-up time (TBUT)	<10 s
Tear osmolarity	TearLab osmolarity	≥ 308 mOsm/L in either eye or interocular difference > 8 mOsm/L
Ocular surface staining	Corneal fluorescein staining	>5 corneal spots
	Conjunctival staining	>9 conjunctival spots
	Lid-margin staining	≥2 mm in length and ≥25% in width

OSDI: Ocular Surface Disease Index; TBUT: Tear film break-up time; mOsm/L: quantitative osmolarity reading

The medical history assessment included potential risk factors such as use of topical/systemic medications, ocular surgeries, dermatologic or systemic inflammatory conditions, lifestyle factors (e.g., smoking, screen exposure), and history of dry eye-related symptoms. Patients were asked about the use of topical corticosteroids, systemic antibiotics, or other MGD treatments within the previous month; severe systemic illnesses; use of medications significantly affecting tear production (e.g., isotretinoin, anticholinergics); and pregnancy or breastfeeding.

### Study instruments and procedures

A full baseline ophthalmologic assessment was conducted, including the OSDI questionnaire, best-corrected visual acuity using a Snellen chart, slit-lamp examination of the anterior segment, Schirmer I test, tear film break-up time (TBUT), vital staining with sodium fluorescein, tear osmolarity using the TearLab® osmometer (TearLab Corporation), intraocular pressure with Goldmann applanation tonometry, and fundus examination. Dry eye tests were performed in a standardized sequence, from the least to the most invasive, as previously recommended [13, 14]. The OSDI questionnaire was completed first, before any ocular examination. Objective assessments were then performed in the following order: tear osmolarity, tear film break-up time, ocular surface staining, and, finally, the Schirmer I test, which was considered the most invasive procedure.

Tear osmolarity was assessed using the TearLab Osmolarity System, a diagnostic device cleared by regulatory authorities and CE-marked as an in vitro diagnostic tool. The system comprises a handheld pen, a single-use microfluidic test card, and a stationary reader. The test card—containing a proprietary 50-nanoliter microfluidic channel with embedded gold electrodes—collects tear fluid via passive capillary action at the lateral inferior tear meniscus. Electrical impedance is measured and converted, via a lot-specific calibration curve, into a quantitative osmolarity reading (mOsm/L) displayed by the reader within seconds. TBUT was performed by applying a fluorescein strip in the inferior fornix, asking the patient to blink 2–3 times, then measure the interval from the last blink to the first dry spot under cobalt-blue illumination. Corneal fluorescein staining was performed by applying fluorescein dye to observe staining defects under cobalt-blue illumination, the cut-off values are reported in Table 1. The Schirmer I test was performed without topical anesthesia using standardized strips placed at the junction of the middle and lateral thirds of the lower eyelid. Patients kept their eyes closed for 5 min, after which the length of strip wetting was recorded in millimeters, reflecting combined basal and reflex tear secretion [13, 14].

To ensure accuracy, both eyes were tested separately using distinct test cards, and the higher osmolarity value was used for diagnostic interpretation. Temperature calibration was obtained by switching the device on at least 30 min before the examination [12]. Three consecutive measurements were taken and averaged to ensure reliability of the readings, as previously reported [15]. The system is quality controlled using reusable electronic check cards daily and control solutions with each test card batch, according to the manufacturer recommendations. Values ≥ 308 mOsm/L are generally considered indicative of dry eye, with 316 mOsm/L often used for moderate-severity thresholds [16]. An inter-eye assessment was performed considering a cut-off difference of 8 mOsm/L between eyes.

The OSDI is a validated 12-item questionnaire widely used to quantify symptoms of DED and their impact on vision-related function and daily activities. It has become a standard outcome measure in both clinical practice and research, supporting diagnosis, severity grading, and monitoring of treatment response. The OSDI assesses three domains—ocular symptoms, vision-related function, and environmental triggers—with each item scored on a 0 to 4 scale ranging from “none of the time” to “all of the time.” [17–19] The total score is normalized to a 0–100 scale, where higher scores indicate greater disease severity. Commonly used cutoffs classify scores as normal (0–12), mild (13–22), moderate (23–32), or severe (33–100) [18–20].

### Treatment protocol

**Fig. 1:**
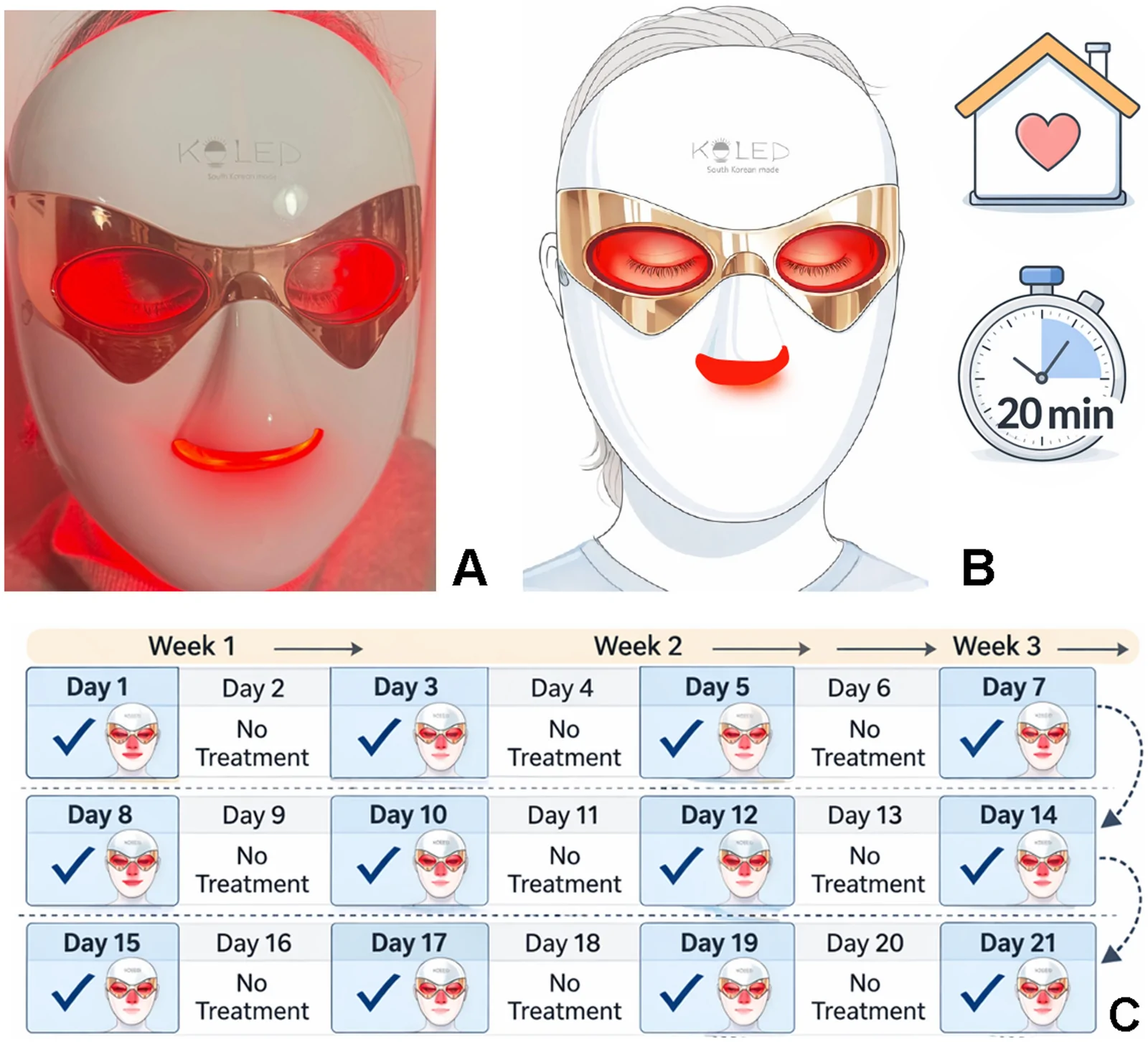
**A** LED Beauty Mask Koled® (Green Remedies S.p.A.) under treatment mode; **B** A treatment session is performed at home, applying the facial mask for 20 min, while the device delivers red light at 630 nm; C, A schematic schedule of treatment sessions on alternate days for 3 weeks

Patients were evaluated before the first session (T0), after 3 weeks of treatment with the LED Beauty Mask Koled® (T1), and 2 weeks after the last session (T2). The device delivers red light at 630 nm and treatment sessions were conducted on alternate days, each lasting 20 min. The device had an approximate retail cost of €600 (approximately US$686) and was not provided by the manufacturer. Patients obtained it independently for cosmetic use, most commonly by renting it from local pharmacies, although some purchased the device. Each mask was used exclusively by a single patient and cleaned in accordance with the manufacturer’s recommendations. The LED Beauty Mask Koled® (Green Remedies S.p.A.) is a home-based, high‑tech LED facial device designed to deliver professional-grade photobiomodulation. It features 576 LEDs operating at four UV‑free wavelengths: (a) green light (520 nm) for calming and soothing superficial skin; (b) yellow light (580 nm) to reduce redness and sensitivity; (c) red light (630 nm) targeting deeper epidermal layers to stimulate skin tone and enhance cosmetic absorption; and (d) near‑infrared (NIR, 850 nm) aimed at deeper dermal tissues to promote collagen and elastin synthesis for improved skin firmness. The mask offers four intensity programs, with NIR illumination active in all modes to maximize anti‑aging effects. Its high LED density and ergonomic design ensure uniform and efficient light coverage while remaining free of ultraviolet and blue light (Fig. 1). Patients applied the mask for 20-min sessions on alternate days over a 3-week period, following the manufacturer’s instructions (https://koled.it/prodotto/high-tech-led-beauty-mask-viso/). The prescribed treatment protocol consisted of 11 home-based sessions performed on alternate days over a 3-week period, with each session lasting 20 min. At the follow-up assessment, patients self-reported high adherence, defined as completion of at least 9 of the 11 scheduled sessions, corresponding to no more than 1 or 2 missed sessions. Patients were asked whether they had experienced significant discomfort or adverse events during treatments. For statistical independence, only one eye per participant was included in the analysis; the study eye was selected at random when both eyes met the eligibility criteria.

### Statistical analysis

Quantitative data were summarized as mean ± standard deviation. Normality was evaluated using Shapiro–Wilk test, considering a *p*-value < 0.05 as a deviation from normality. Changes in quantitative variables before and after treatment were analyzed using paired t-tests or Wilcoxon signed-rank tests when normality assumptions were violated. To compare quantitative parameters at different time points, T0: baseline, T1: 3-weeks, and T2: 2- weeks after last treatment, a repeated measures analysis of variance (ANOVA) was performed. Mauchly’s test of sphericity was used to assess sphericity. If the assumption was violated (*P* < 0.05), Greenhouse–Geisser correction was then applied. A two-sided *p*-value threshold of 0.05 was considered statistically significant. All statistical analyses were conducted using RStudio software (version 2022.07.1; RStudio, PBC, Boston, MA, URL: http://www.rstudio.com/).

## Results

A total of 20 eyes of 20 patients (20 females) with moderate DED and MGD were analyzed. All enrolled participants were postmenopausal women. Best-corrected visual acuity was 20/20 in all the eyes included in the study. Baseline characteristics are reported in Table 2.

**Table 2:** Baseline characteristics of the study population

Variables	Patients (n=20)
Age	63.4 ± 3.2 years
Gender, females	20 (100%)
OSDI score	19.3 ± 6.7
Schirmer test	20.6 ± 9.4
TBUT	7.6 ± 1.4
mOsm/L	312 ± 16.7

OSDI: Ocular Surface Disease Index; TBUT: Tear film break-up time; mOsm/L: quantitative osmolarity reading

At baseline, corneal fluorescein staining was observed in 6 eyes (30%), but this finding was no longer evident at the final follow-up.

Longitudinal changes in dry eye parameters are presented in Table 3. Overall treatment adherence was 99.5%, with 219 of 220 planned sessions completed; only one patient missed a single session.

**Table 3:** Longitudinal changes in objective tear film parameters and OSDI scores following treatment

	Baseline	T1	T3	F	*P* value
Schirmer I	20.6 ± 9.4	21.2 ± 9.3	17.8 ± 6.8	1.26	0.29
TOsm	314.8 ± 14.6	286.9 ± 4.7	287.3 ± 4.7	32.3	<0.001
TBUT	7.6 ± 1.4	10.4 ± 2.1	10.3 ± 1.15	14.4	0.001
OSDI	17 ± 4.1	10.6 ± 4.8	8.8 ± 3	52.1	<0.001

TBUT: Tear film break up time; TOsm: Tear Osmolarity mOsm/L; OSDI: Ocular Surface Disease Index

Tear osmolarity showed a significant improvement over time (*P* < 0.001), a post-hoc analysis revealed a significant difference between T0–T1 (mean difference: 27.9 ± 3.9, *p*<0.001) and T0-T2 (mean difference: 27.5 ± 3.9, *p*<0.001).

Furthermore, four eyes (20%) exhibited osmolarity values below the measurable range, suggesting normalization of tear composition or lower-limit measurement effects.

Tear film stability, assessed by BUT, improved significantly following treatment (*P* = 0.001). The post-hoc analysis demonstrated that the significant change was between T0-T1 (mean difference: −2.8 ± 0.6, *p*<0.001) and T0–T2 (mean difference: −2.7 ± 0.6, *p*<0.001), while no significant differences were detected between T1–T2 weeks post-treatment (mean difference: 0.08). Subjective dry eye symptoms, measured using the OSDI, also demonstrated a significant and sustained reduction over the study period (*P* < 0.001). In contrast, no significant changes were observed in aqueous tear production as measured by the Schirmer I test (*P* = 0.29).

At the end of treatment, the patients reported no discomfort or other adverse events associated with the procedure.

## Discussion

This study reported the effects of the commercial LED Beauty Mask Koled®, originally designed for dermatological disorders, on moderate DED. The home-use device shares the same technical specifications as the patented ophthalmic device Meibomask®, emitting red light at 630 nm and near infrared light at 850 nm (https://koled.it/prodotto/high-tech-led-beauty-mask-viso/). This wavelength has been postulated to be predominantly absorbed by mitochondria, increasing the activity of cytochrome c oxidase (COX) in the electron transport chain. Therefore, photobiomodulation works primarily by restoring mitochondrial function through photon absorption by COX, leading to improved energy metabolism and activation of multiple signaling pathways that promote tissue repair, reduce inflammation, and enhance cell survival. [21] LLLT treatment in eyes with MGD has been shown to improve fluorescein corneal staining (FCS) score, lissamine green conjunctival staining (LGCS) scores, Schirmer’s test, and upper meibography after 3 weeks of treatment twice a week compared with placebo [22]. In our series, despite the mixed changes in Schirmer I values, improvements in both TOsm and BUT suggest an overall enhancement in tear film stability and ocular surface condition following treatment. Tear osmolarity demonstrated a strong association with disease severity, with higher values generally observed in more severe disease [12]. Notably, in our study population, the changes in tear osmolarity were among the most compelling findings. The greatest change in TOsm was evident three weeks after treatment initiation, supporting a possible direct effect of LLLT on tear osmolarity, as previously postulated [23]. Although MGD is not necessarily the primary driver of changes in tear osmolarity, this parameter has demonstrated higher sensitivity than corneal and conjunctival staining and meibomian gland grading, as well as higher specificity than tear break-up time and Schirmer testing, with a strong correlation to DED severity [24]. Another important factor to consider in our cohort is that all participants were postmenopausal women, a population known to exhibit altered tear osmolarity. Tear osmolarity has been shown to be directly associated with sex hormone levels, suggesting a causal link between hormonal changes and the development of dry eye in postmenopausal women [25]. This observation has dual relevance, as LED-based devices are widely used in dermatology and medical aesthetics for skin rejuvenation, with particularly high utilization among postmenopausal women [26, 27]. Given the potential influence of light-based therapies on tear film homeostasis and dry eye parameters, this overlap may be especially relevant in postmenopausal women, who are already at increased risk of dry eye disease, and may help inform clinical recommendations regarding the use of facial LED treatments in patients with underlying ocular surface disorders.

Several studies have investigated combination therapy with IPL and LLLT, demonstrating overall improvements in clinical parameters of DED [28–33]. More recently, a few studies have explored LLLT as a stand-alone treatment, employing different light parameters, such as infrared light (e.g., Lutronic HEALITE II; 830 nm wavelength; 100 mW/cm^2^ irradiance, lutronic.com) or lower irradiance red light (e.g., Espansione Group My-mask device; 625 nm wavelength; 35 mW/cm^2^ irradiance, espansionegroup.it) [7–9, 22]. These studies reported improvements in OSDI score, non-invasive tear breakup time (NIBUT), tear meniscus height (TMH), tear film lipid layer thickness, and Schirmer’s test results. Furthermore, LLLT has been shown to induce molecular changes, including reductions in interleukin-1β, interleukin-17F, MMP-9, MMP-9/TIMP-1 ratio, and ocular surface B-cell proportions. This downregulation of proinflammatory mediators supports its therapeutic effect in patients with DED and MGD [31].

In a recent study, a novel home-use mask was evaluated, showing improvements in non-invasive BUT, TMH, and OSDI scores. The home-use device offers several advantages. It is fully independent, requiring no additional hardware or software, and is designed as a user-friendly “plug and play” system that can be operated without technical expertise by either clinicians or patients. The procedure itself is painless and provides immediate symptomatic relief [8].

Although the overall observation period was limited to 5 weeks, the timing of the improvements in tear osmolarity, TBUT, and OSDI is consistent with previous studies reporting improvements in tear film stability, ocular surface indices, and subjective symptoms after LLLT in a range comprised between 2–4 weeks [4, 7–10].

In the present study, significant improvements were detected immediately after the 3-week treatment period and remained evident 2 weeks after the final session. The persistence of these changes at two consecutive time points may further support the reliability of the findings and the possibility of an early improvement in tear film composition and stability. Whether these benefits are reproducible and persist over the long term remains to be determined.

While the results of this study are encouraging, several limitations must be acknowledged. First, this was a retrospective cohort and was not intended or powered to function as a clinical trial. Furthermore, the absence of a control group makes it difficult to distinguish the therapeutic effects of the LED mask from placebo effects or natural fluctuations in dry eye severity. Second, the study included a small, all-female sample of postmenopausal women, which limits the generalizability of these clinical findings to broader and more diverse populations, including male patients. Third, variability in adherence to the home-based treatment protocol could influence outcomes, and no objective measures of compliance were collected. Finally, the relatively short follow-up period precluded assessment of long-term durability and the potential need for a structured maintenance therapy protocol.

Furthermore, although the Koled® mask shares technical specifications with ophthalmic devices, it was originally developed for dermatological purposes, and its optimal treatment parameters for ocular use (wavelength combinations, irradiance, and exposure time) remain to be validated.

Despite these limitations, the potential of home-based LLLT remains significant. Compared with in-office professional devices, home-use systems are more accessible, cost-effective, and convenient, allowing patients to undergo frequent treatments without the logistical and financial barriers of clinical visits. This accessibility could enhance adherence and expand therapeutic options for individuals with dry eye disease and meibomian gland dysfunction, particularly in resource-limited settings. Moreover, built-in safety mechanisms such as preset protocols and session limits support safe self-administration. If validated in well-designed, randomized controlled clinical trials, home-use devices may become a valuable adjunct or alternative to office-based therapies, offering a scalable and patient-centered strategy for managing ocular surface disease.

## Data Availability

No datasets were generated or analysed during the current study.
